# Trials recruiting patients in the acute NHS setting: trial management challenges

**DOI:** 10.1186/1745-6215-16-S2-O47

**Published:** 2015-11-16

**Authors:** Seonaidh Cotton, Ruth Thomas, Sarah Cameron, Anne Duncan, Tracey Davidson, Kirsty McCormack

**Affiliations:** 1 University of Aberdeen, Aberdeen, UK

## 

There are challenges to conducting randomised controlled trials: some are common across trial settings; others relate to the setting itself. We present some challenges for publically-funded trials recruiting within the acute NHS setting. We draw on experience from three such RCTs: SCARLESS (Single Port/Incision Laparoscopic Surgery versus standard laparoscopic surgery for appendicectomy); SUSPEND (alpha blockers, calcium channel blockers versus placebo for urinary stones) and NIAMI (intravenous sodium nitrite versus placebo in patients with acute heart attack).

Across all three studies, patients presented out-of-hours. To facilitate out-of-hours recruitment, the wider clinical team identified eligible patients, provided study information, obtained consent and recorded data without the support of a research nurse. In some cases, they also delivered the intervention. Training the wider clinical teams to undertake these duties was resource-intensive, particularly because of junior staff rotation.

Again, across studies, there were challenges in intervention delivery. In SCARLESS there was tension around randomising patients: a senior surgeon provided cover for the novel surgery, but junior surgeons were keen to develop their skills by undertaking standard surgery. In SUSPEND, recruitment was constrained to the opening hours of clinical trials pharmacies to dispense study medication. This particular challenge was addressed in NIAMI by the study team holding study medication, even for patients presenting in-hours.

A further, and more unexpected challenge was in relation to engaging participants in follow-up. Retention to primary outcome was challenging; perhaps because patients have not had a “chronic” condition and have “moved-on” from the event that brought them into the trial.

